# Clinical outcome and implant accuracy in cervical fractures stabilization using 3D-printed patient-specific drill guides for placement of bicortical screws in 16 dogs

**DOI:** 10.3389/fvets.2026.1850379

**Published:** 2026-10-15

**Authors:** Alba Manzanedo-Delgado, Bill Oxley, Sebastien Behr

**Affiliations:** 1 Willows Veterinary Centre and Referral Service, Solihull, United Kingdom; 2 Vet 3D Billy’s Space, Cumbria, United Kingdom

**Keywords:** 3D-printed patient-specific drill guides, canine, cervical vertebral fracture, dog, neck fractures, spinal stabilization

## Abstract

**Objective:**

To describe the clinical outcome of dogs with cervical fractures undergoing spinal stabilization surgery using 3D-printed patient-specific drill guides, and to evaluate the accuracy of pedicle screw placement with this technique.

**Study design:**

Retrospective descriptive study.

**Animals or sample population:**

Sixteen dogs.

**Methods:**

Medical records were searched between August 2015 and January 2025 for dogs with traumatic spinal fracture via CT scan causing signs of cervical myelopathy that underwent spinal stabilization surgery using 3D-printed patient-specific drill guides followed by a postoperative CT scan. Neurological status was graded retrospectively with the Modified Frankel Score (MFS) on presentation, preoperatory on the day of the surgery, postoperatively on the first day after surgery, at hospital discharge and at the follow-up examination performed 4–6 weeks after surgery, when available. A telephone questionnaire was obtained for the long-term outcome.

**Results:**

Sixteen dogs met the inclusion criteria (3/16 grade 2 MFS, 5/16 grade 3 MFS, 7/16 grade 4 MFS and 1/16 grade 5 MFS). Two dogs showed mild minor transient intraoperative complications. Optimal pedicle screw placement was achieved in 85/90 (94.4%) of screws. Twenty-four hours after surgery, the neurological grade remained unchanged in 10/16 dogs, improved in 5/16, and transiently deteriorated in 1/16 dog, which recovered by discharge. Postoperative complications occurred in 10/16 dogs and resolved with medical management in 9/10 cases. Short term follow-up revealed a static stable (6/14) or improved (8/14) neurological grade since discharge. Among the 12 dogs with owner-reported long-term follow-up, all were reported by their owners to be ambulatory and to have improved neurologically. Long-term quality-of-life (5 = excellent; 1 = poor) was scored by the owners 5/5 in 11/12 (91.7%) dogs and 4/5 in 1/12 (8.3%) dogs.

**Conclusion:**

Dogs in this study overall had an improved neurological status and favorable long-term outcomes when follow-up information was available. However, postoperative adverse events, including complications, should be considered when interpreting overall clinical outcome.

## 1 Introduction

Vertebral fractures and luxation in dogs typically result from severe external trauma, most frequently associated with road traffic accidents (RTAs), accounting for 40–60% of cases. However, other frequent causes of injury have been reported, such as animal attacks or falls from heights (1–4). Due to the nature of these injuries, studies have indicated that 48–63% of affected animals may have concurrent injuries, including fractures of long bones, mandible, scapula, occiput, and thoracolumbar vertebrae (2).

The distribution and incidence of vertebral column injuries have been previously classified, with the thoracolumbar vertebrae being the most frequently affected, followed by the lumbar, cervical, sacrococcygeal and most uncommon thoracic vertebrae (1, 2).

Within the cervical region (C1-C7), veterinary studies have shown that fractures of the body of the axis (C2) account for approximately 50% of all cervical fractures (2). This finding aligns with human studies, where fractures of the axis constitute nearly 20% of all acute cervical spinal fractures (5). Other commonly affected areas in dogs include dens fractures of C2, often caused by high-speed trauma and secondary forcible flexion of the neck, and fractures of the atlas (C1) with an incidence of up to 25% in cervical fractures; where approximately 34% of cases, multiple cervical vertebrae are affected, with the C3, C4, and C5 being overrepresented. In contrast, single vertebra fractures occur in only about 15% of cases (3).

The management of vertebral fractures has long been a topic of debate, with approaches divided between conservative and surgical interventions (1). The primary objective of treatment is to mitigate further damage to neural structures and alleviate compression, if present. As such, the degree of spinal instability is a critical determinant in selecting the appropriate treatment strategy. Conservative management, including external immobilization with splints or casts, may be associated with complications such as device loosening, skin abrasions, pressure ulcers, pain, and exacerbation of instability, potentially contributing to neurological deterioration (2–4). Several surgical stabilization techniques have been described for the management of cervical vertebral fractures in dogs, including the use of pins, plates, screws-based constructs, such as bicortical and transpedicular (pedicle screw) fixation, often combined with polymethylmethacrylate (PMMA), with the choice of construct depending on fracture configuration, vertebral anatomy, and surgeon preference (2–4). Among these techniques, ventral stabilization using bicortical screws and PMMA is one of the most commonly reported methods for restoring spinal stability in dogs with cervical fractures (1, 4).

Patient-specific three-dimensional (3D)-printed surgical guides have gained increasing interest in veterinary surgery because of their potential to improve surgical precision and implant positioning across a range of procedures (6, 7). More recently, the incorporation of three-dimensional (3D) patient-specific drill guides has been introduced to improve the accuracy of implant placement during spinal stabilization (8–16).

Multiple veterinary studies have demonstrated high pedicle screw placement accuracy using 3D-printed patient-specific drill guides (3D-PSGs), including applications in the cervical (8–11), thoracolumbar (11–14), and lumbosacral vertebral column (15, 16) in dogs, supporting the accuracy and feasibility of this technique. However, although cervical applications of 3D-PSGs and screw placement accuracy have been previously reported (8–11), veterinary literature specifically evaluating their clinical application in dogs with traumatic cervical vertebral fractures remains scarce, particularly regarding perioperative outcomes and the potential procedural advantages associated with patient-specific navigation.

Similarly, a recent study comparing individualized 3D-printed navigation templates with conventional freehand pedicle screw placement for upper cervical fractures reported higher screw placement accuracy (95.7% versus 80%), together with shorter surgical times, improvement in pain management, reduced blood loss and decreased fluoroscopy requirements (17). Similar improvements in pedicle screw placement accuracy have also been reported in other human studies evaluating 3D-printed navigation-assisted techniques (18, 19). Although these findings cannot be directly extrapolated to dogs, they provide relevant background regarding the potential advantages of patient-specific navigation technologies.

To the authors’ knowledge, this is the first retrospective study to evaluate both the short- and long-term outcomes of dogs with cervical fractures treated with cervical stabilization using 3D-PSGs.

The aim of this retrospective, descriptive study was to evaluate the accuracy of screw placement, short and long-term clinical outcomes of dogs with cervical fractures treated by vertebral stabilization using 3D-PSGs for pedicle screw placement.

## 2 Materials and methods

### 2.1 Inclusion criteria

Medical records for dogs with cervical (C1-C7) vertebral fracture from a single institution (Willows Veterinary Centre and Referral Service) were retrospectively reviewed between August 2015 and January 2025. All dogs with pre and immediate postoperative computed tomography (CT) data that had undergone segmental vertebral stabilization with the aid of 3D-PSGs were included in the study. Surgical stabilization was recommended based on the presence of cervical vertebral fractures considered clinically unstable by the attending surgeon following assessment of fracture configuration, vertebral displacement, and associated luxation (Supplementary Table S1). All surgeries were performed by a European College of Veterinary Neurology (ECVN) Diplomate with the assistance of an ECVN resident.

Collected data included signalment (breed, age, sex and body weight), neurological function and diagnostic imaging findings. Neurological function was graded retrospectively using the Modified Frankel Score (MFS) based on information extracted from contemporaneous neurological examinations documented in the medical records. MFS grades were reconstructed retrospectively by the authors rather than prospectively assigned during clinical management. MFS was assessed with grade 1 for tetraplegia with no superficial nociception, grade 2 for tetraplegia with intact superficial nociception, grade 3 for non-ambulatory tetraparesis, grade 4 for ambulatory tetraparesis with ataxia and grade 5 for cervical spinal hyperesthesia only (20). MFS was retrospectively reconstructed from contemporaneous medical records at each of the following time points: at presentation, preoperatively on the day of surgery, on the first postoperative day, at discharge, and at the short-term follow-up appointment when available. Availability of short- or long-term follow-up was not required for study inclusion. Cases lacking follow-up information were retained in the study if preoperative and immediate postoperative data met the inclusion criteria. Consequently, the completeness of follow-up data varied across the study population, as summarized in Figure 1. The use of a neck brace during the preoperative period and the occurrence of intraoperative, perioperative and postoperative complications were recorded for each case. Complications were categorized according to their timing as intraoperative (occurring during general anesthesia and the surgical procedure), immediate perioperative (within the first 12 h following surgery), postoperative (>12 h after surgery until the short-term follow-up examination at 4–6 weeks), and long-term (thereafter, assessed using a standardized owner telephone questionnaire). Complications were retrospectively classified as minor, major or catastrophic according to the criteria proposed by Cook et al. (21)

**Figure 1:**
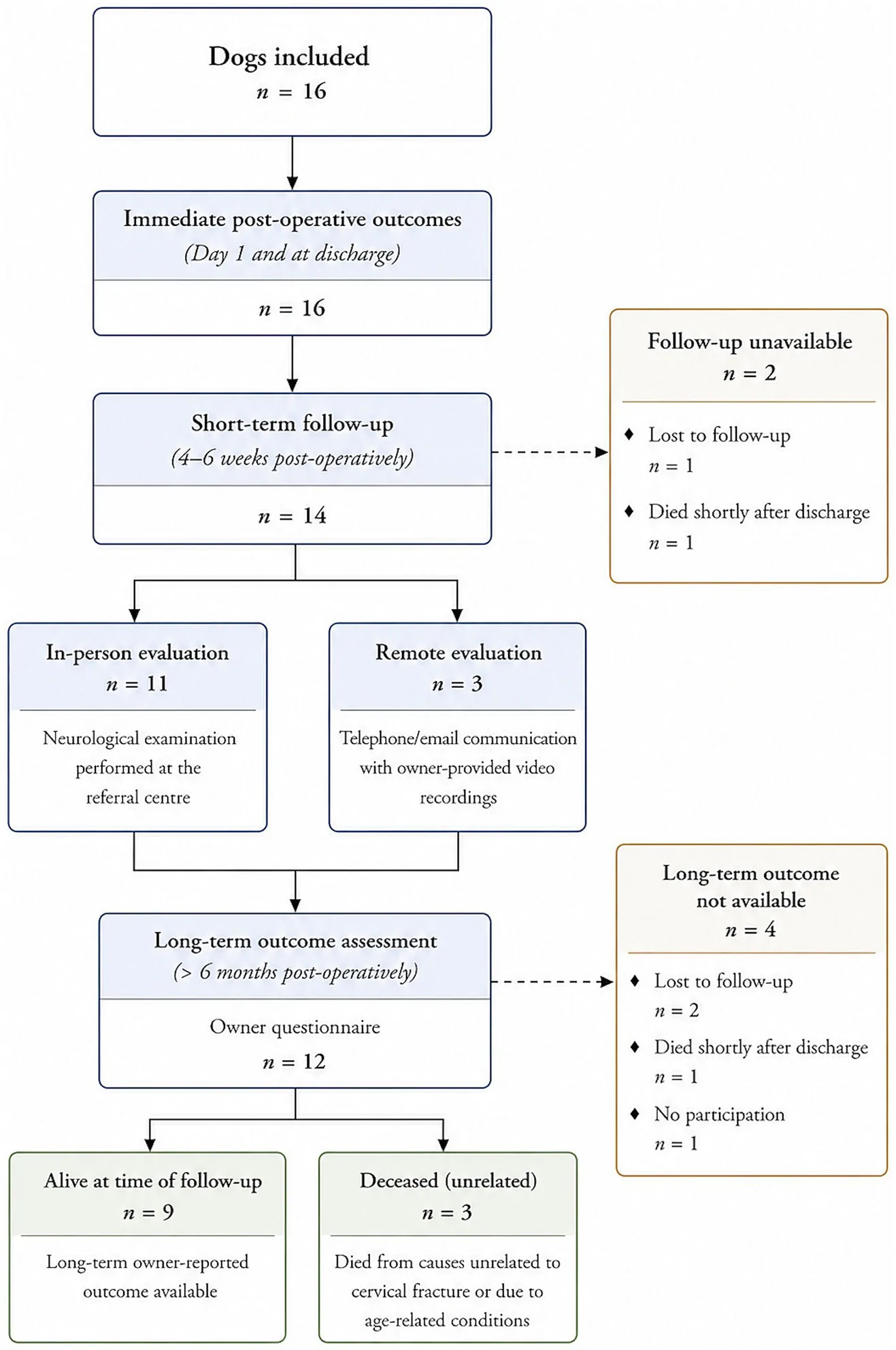
Flow diagram of follow-up availability and data collection. Numbers represent the number of dogs with data available at each time point.

Dogs were excluded if they did not undergo cervical fracture stabilization using 3D-printed patient-specific drill guides, lacked the required CT scan examinations post-operatory, or did not otherwise fulfil the inclusion criteria.

### 2.2 Production of the drill guides

Computed tomography images of the cervical spine were acquired (0.6-mm thick; GE BrightSpeed scanner; General Electric Medical Systems), and the resultant Digital Imaging and Communications in Medicine (DICOM) images were exported into medical image processing software (Osirix; Pixmeo SARL, Geneva, Switzerland).

The term “pedicle screw” is used throughout the manuscript to refer to screws (cortical or locking screws) inserted through the vertebral pedicle and not to a specific implant type. A 3D virtual model of each vertebra was then created using computer aided design software (Netfabb Professional, Netfabb GmbH). Each 3D vertebral virtual model could be individually manipulated in all planes to allow precise planning of each pedicle screw trajectory, using a virtual cylinder of equal diameter to the screw being placed. The planned trajectories were selected to maximize bone purchase, including bicortical engagement where anatomically appropriate, while maintaining a safe distance from critical structures such as the vertebral canal, spinal cord, nerve roots, and transverse foramina, according to the optimum implantation corridors previously described in dogs (22). The screw trajectories were planned by two of the authors. The screw diameter and length chosen based on the dimensions of the pedicle and/or vertebral body, aiming for a screw diameter to pedicle width ratio greater than 75%. A virtual drill guide was then created for each vertebra as previously described (9). Drill guide sleeves were created around the planned trajectories with an internal diameter corresponding to that of the drill bit used for the proposed pilot hole. The size of the pilot hole was dependent on the screw diameter chosen according to manufacturer’s recommendations. The contact surface of the drill guide was constructed as an inverted representation of the ventral vertebral surface such that the finished guide would fit onto the cortex in a unique position (Figure 2). Both the vertebrae (with the pilot holes) and the drill guides were 3D-printed in methacrylate photopolymer resin (BioMed Amber resin; Figure 3). The resin used to print the 3D printed drill guides was certified as both biocompatible and autoclavable (ISO Standard for Formlabs Vertex-Dental BV resin: EN-ISO 10993-1:2009/AC: 2010, USP Class VI, Utrecht, The Nether- lands). The interval between CT acquisition and surgery, including drill guide design, manufacture and delivery, was between 2 to7 days. Prior to surgery, the guide fit was checked for each guide on the vertebral model, and the guides and vertebral models were steam sterilized (134c for 20 min).

**Figure 2:**
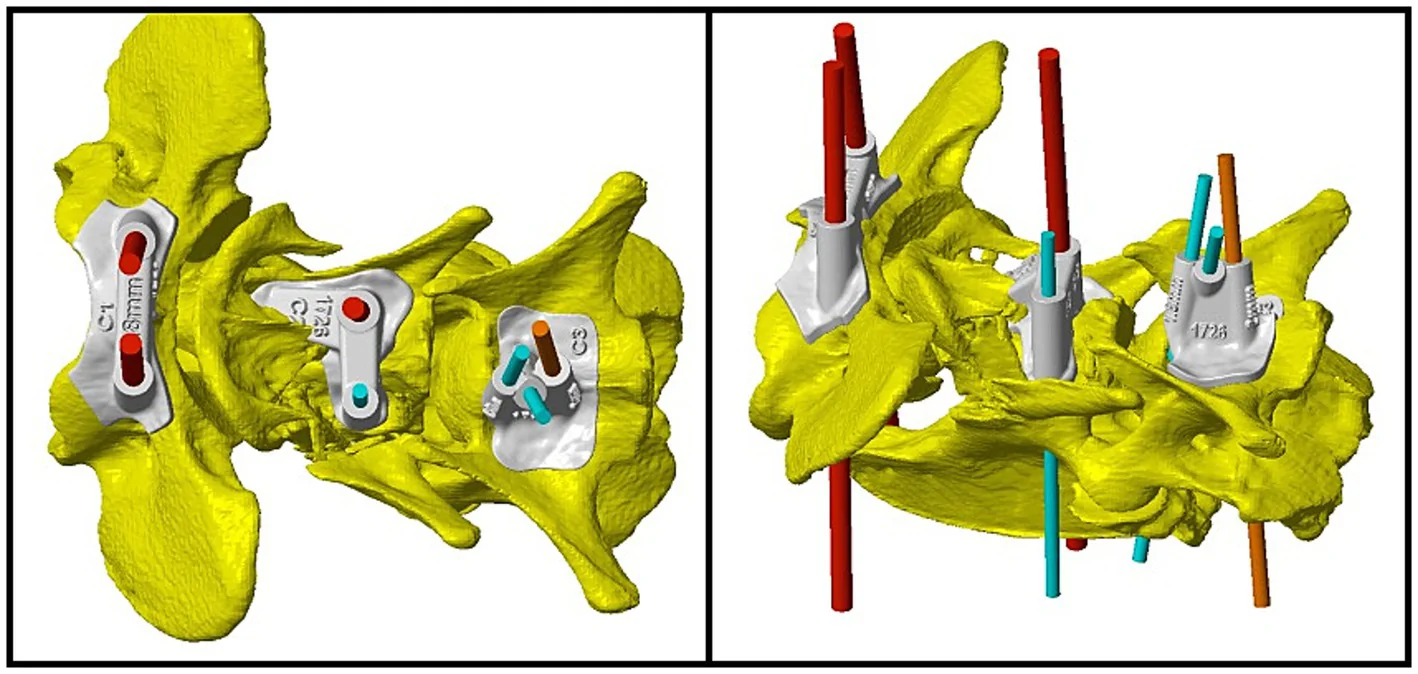
Computer-aided design images displaying drill guides *in situ* at C1, C2, C3 and planned trajectories in a dog with a C2 fracture.

**Figure 3:**
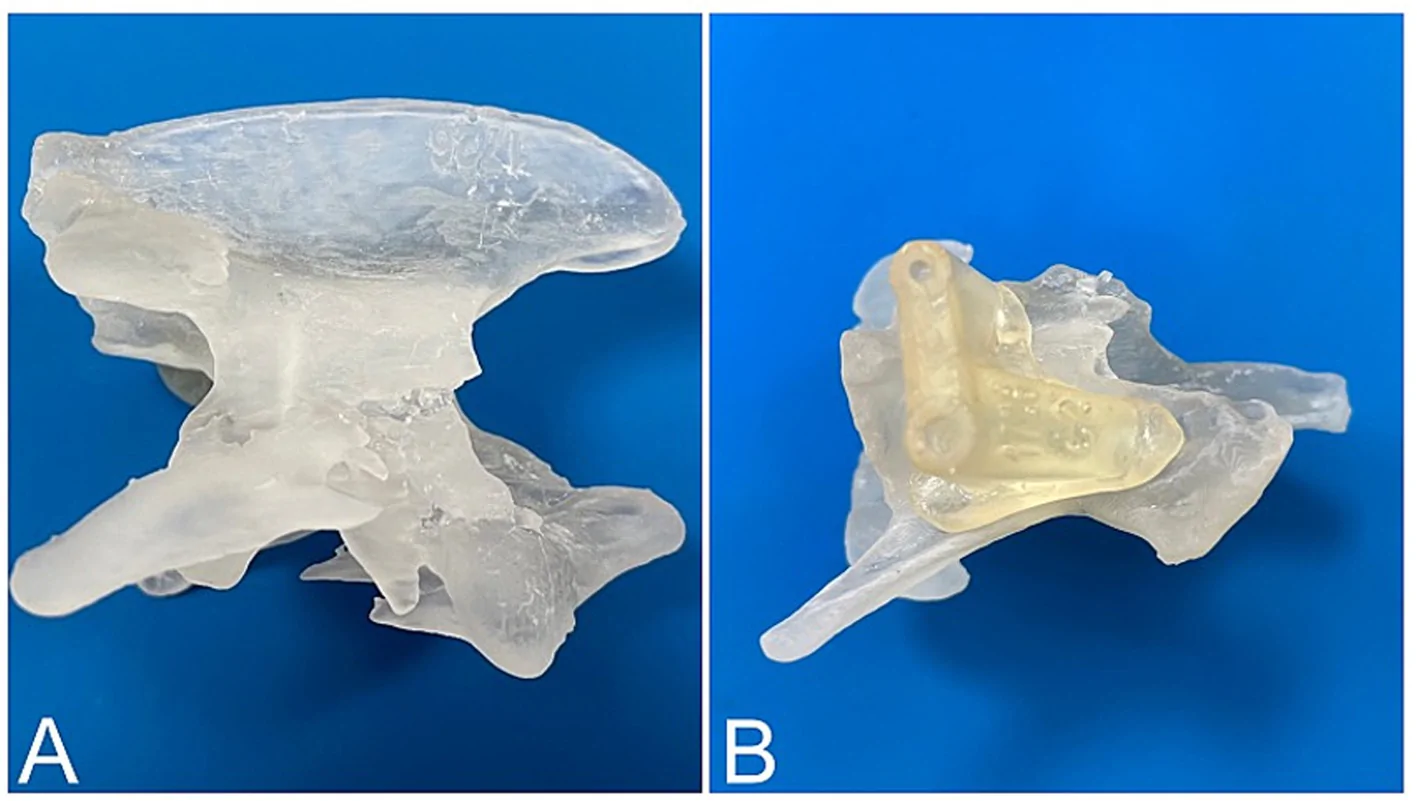
3D printed resin model of C2 comminuted fracture **(A)** and ventral view of C2 with 3D printed drill guide *in situ* **(B)**.

### 2.3 Surgical technique and screw rating

All patients were positioned in dorsal recumbency for a ventral approach to expose the ventral surface of the vertebrae to be stabilized. Once the area of the 3D-PSG footprint on each vertebra was exposed and cleared from soft tissue, the 3D-PSG were placed to drill the pilot holes sequentially for each vertebra. All pilot holes were drilled using 3D-PSG. Once all the pilot holes were drilled, the drill guide was removed, and the screws were placed in each vertebra. The vertebral stabilization of the affected vertebrae was then performed by bonding the screw heads with bone cement. Subject to pedicle size, each cervical vertebra was instrumented with between 2 and 4 pedicle screws.

Care was paid in positioning the patient with the neck in extension due the unstable cervical fractures. The position was maintained during the surgery by securing the dog with tape and rope ties to the table. The extend of the midline approach varied depending on which cervical vertebrae required exposure. The cranial cervical vertebral column (C1 and C2) was approached via a ventral midline approach; this involved transection of the right sternothyroideus muscle and retraction of the larynx to the left. Once the vertebras of interest were exposed, any residual soft tissues on the ventral cortices, in the area of the guide footprints, were removed using both a periosteal elevator and a scalpel blade to allow precise fit of the guide to the ventral vertebral surface. With the drill guides in place (Figure 4), bicortical pilot holes were drilled as indicated. The pilot hole size was determined by the screw size chosen (e.g., a 1.1 mm drill bit for a 1.5 mm screw) according to manufacturer recommendations. Bicortical stainless steel or titanium self-tapping screws were placed. The screw diameter chosen (from 1.5 to 3.5 mm) varied depending on the pedicle size. For screw diameter above 2.0 mm, locking screws were used for their greater core diameter in comparison to an equivalent cortical screw.

**Figure 4:**
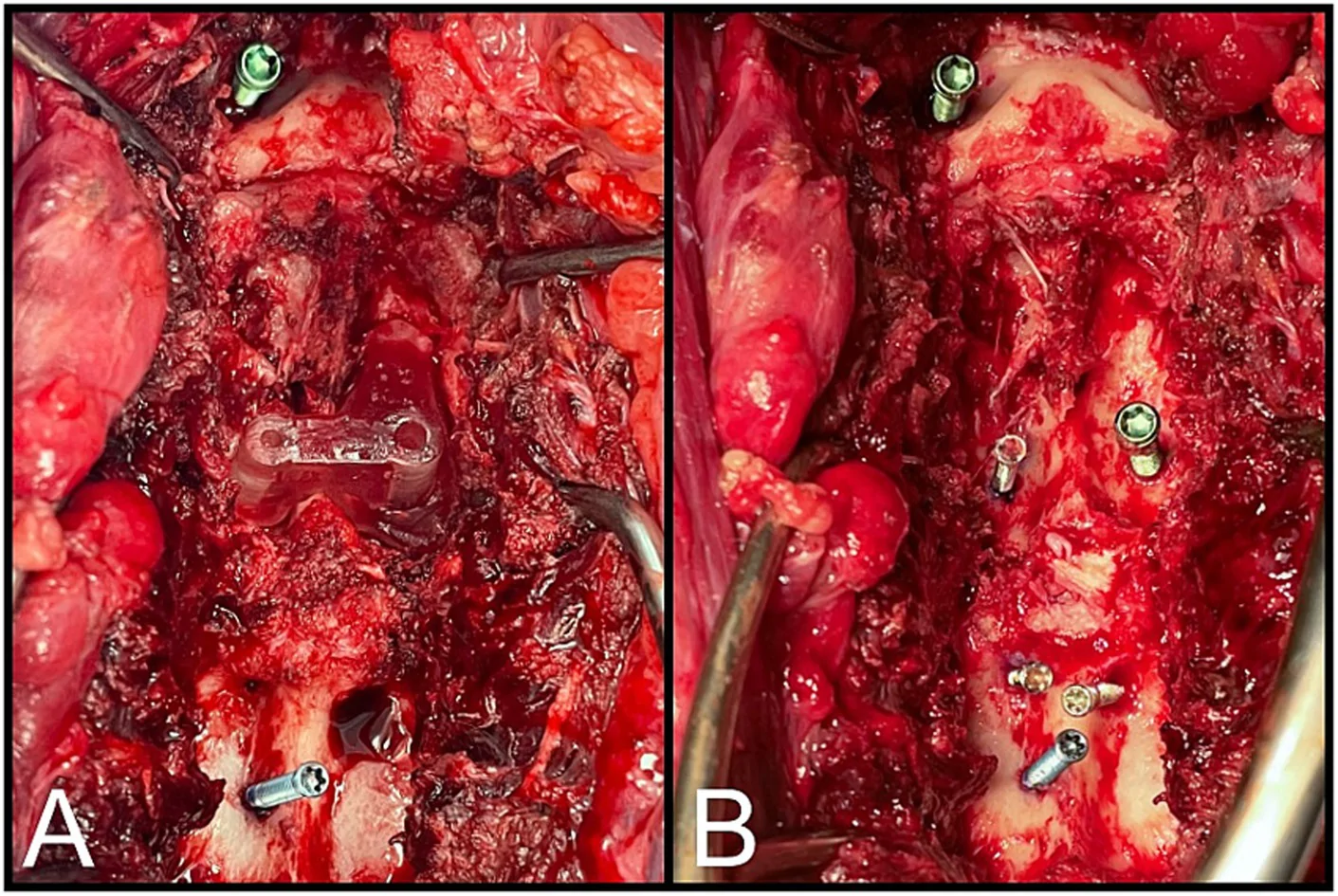
Intraoperative photographs displaying C2 3D-PSGs model **(A)** and the result following screw placement **(B)**.

The ventrally projecting screw ends were left protruding from the cortex (typically between 5-10 mm) for bonding with PMMA cement (Heraeus, Palacos, R + G, Zimmer Biomet, Winterthur, Switzerland). The length of each screw left protruding varied depending on patient size and affected vertebrae; and was adjusted per-operatively not to protrude over the longus coli muscles. The volume of bone cement applied varied depending on patient size to allow all screw heads to be incorporated in the construct. Copious irrigation was performed to minimize tissue injury during the exothermic curing process. Closure of the muscle and skin layers was routine.

CT of the cervical vertebral column was performed immediately after surgery. Screw position grading was initially performed by one author (A Manzanedo-Delgado) based on the postoperative CT scan in which the data were reformatted such that each screw was visible along its long axis, permitting assessment of any breach of the screw across the adjacent pedicular cortices (Figure 5). The observer was blinded to clinical outcomes at the time of image assessment. All grading results were subsequently reviewed by the board-certified neurologist, SB.

**Figure 5:**
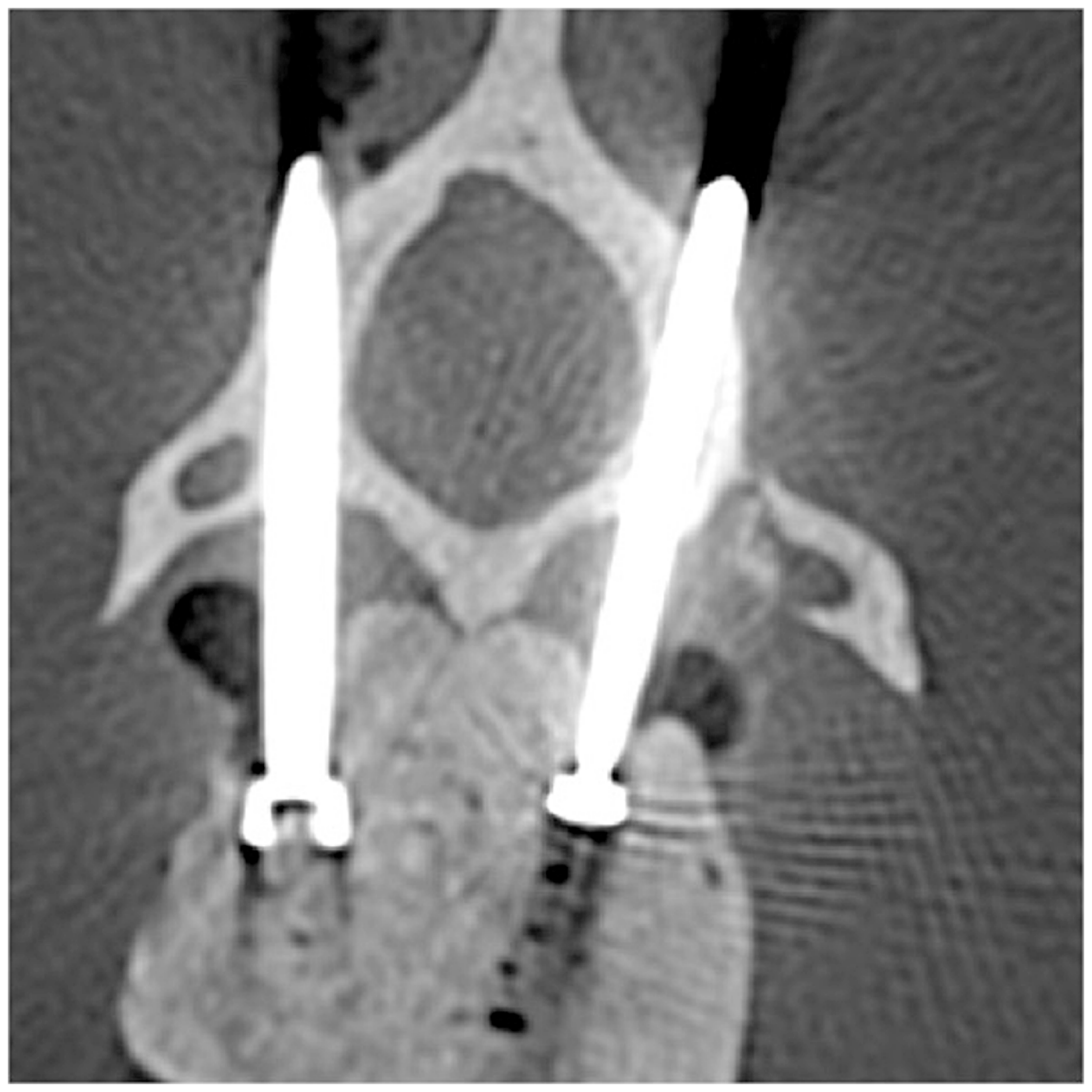
Transverse computed tomographic image postoperatively with well positioned bicortical screws in C2.

Screws were subjectively graded using a Zdichavsky modified classification (Figure 6) (23). ‘Grade 1’ was given if the screw was fully contained within the pedicle and vertebral body, ‘Grade 2a’ if there was penetration of the medial pedicle wall and ‘Grade 2b’ if the screw was entirely medial to the pedicle wall.

**Figure 6:**
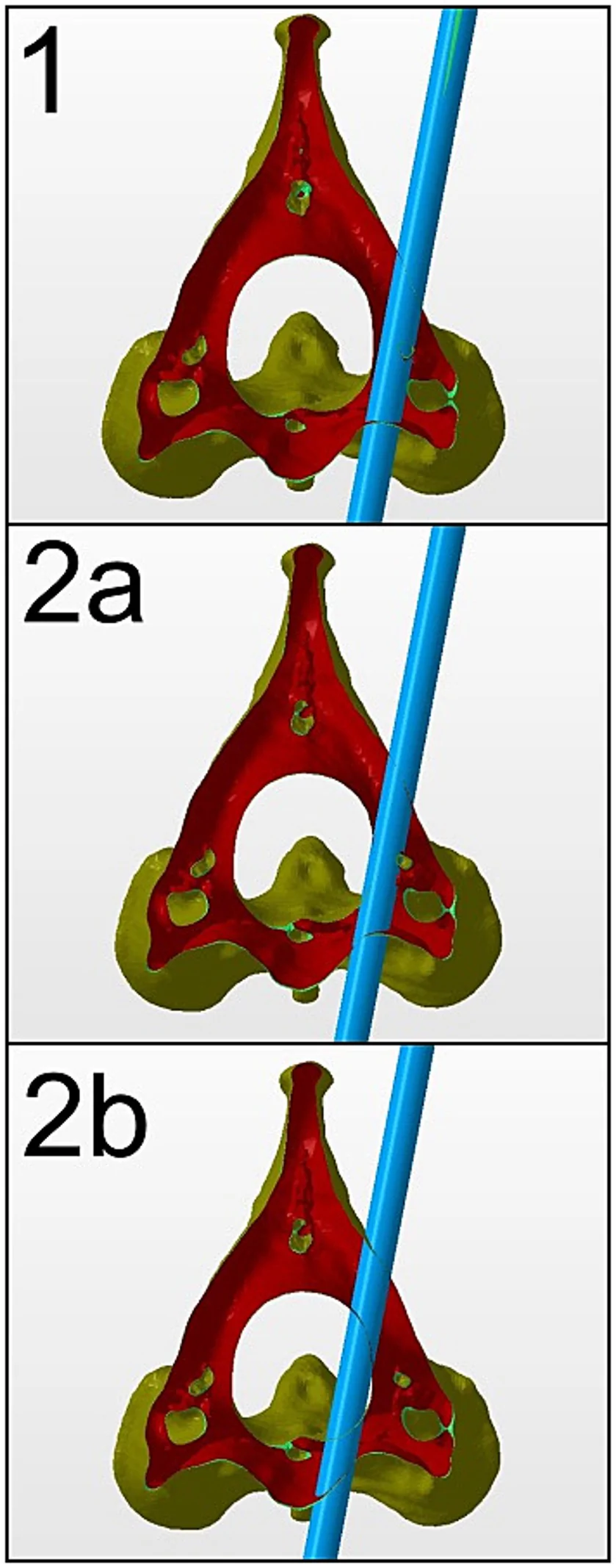
Screw grading Transverses images of C2 with screws in grade 1, 2a, 2b.

### 2.4 Short-term outcome

Assessment of short-term outcomes was conducted through a retrospective review of medical records, focusing on the identification of perioperative complications and the evaluation of neurological status (MFS grade) on the first postoperative day and at the time of hospital discharge. Follow-up data obtained during re-examinations at the referral institution when available, typically performed between four and six weeks postoperatively (Table 1). Neurological progression was classified as improvement (a decrease of at least one MFS grade), stable (no change in MFS grade), or deterioration (an increase of at least one MFS grade) relative to the preoperative assessment. Additionally, the presence of neck brace during the preoperatory period and the assessment of neck pain on the first follow-up (between 4 to 6 weeks postoperatively) was also recorded (Table 2).

**Table 1:** Summary clinical data, complications, accuracy of screw placement, and short-term outcome.

Patients number	Signalment (breed, age and body weight)	Complications	Screws grading score	Neurological score
1	English Pointer, 9 months, 27 kg	Preoperative: None Intra−/perioperative: None Postoperative: Edema front paws	Score 1: 4 Score 2a: 0 Score 2b: 0	At Admission: 5 Preoperative: 5 Day 1 postoperative: 4 Discharge (2 days postoperative): 5 Follow-up: Normal
2	Pug, 5 years, 7.6 kg	Preoperative: regurgitations Intra−/perioperative: None Postoperative: regurgitations	Score 1: 4 Score 2a: 0 Score 2b: 0	At Admission: 3 Preoperative: 3 Day 1 postoperative: 3 Discharge (3 days postoperative): 3 Follow-up: 4
3	Chihuahua, 9 months, 3.6 kg	Preoperative: None Intra−/perioperative: None Postoperative: None	Score 1: 3 Score 2a: 1 Score 2b: 0	At Admission: 4 Preoperative: 3 Day 1 postoperative: 4 Discharge (4 days postoperative): 4 Follow-up: Normal
4	Goldendoodle, 5 years 5 months, 16 kg	Preoperative: None Intra−/perioperative: None Postoperative: None	Score 1: 8 Score 2a: 0 Score 2b: 0	At Admission: 3 Preoperative: 3 Day 1 postoperative: 4 Discharge (4 days postoperative): 4 Follow-up: 5
5	Toy Poodle, 7 months, 2.8 kg	Preoperative: None Intra−/perioperative: None Postoperative: Constipation, regurgitation, gaging and reaching.	Score 1: 5 Score 2a: 0 Score 2b: 0	At Admission: 3 Preoperative: 3 Day 1 postoperative: 3 Discharge (12 days postoperative): 4 Follow-up: Normal
6	Dalmatian, 6 years, 27.5 kg	Preoperative: None Intra−/perioperative: None Postoperative: None	Score 1: 6 Score 2a: 2 Score 2b: 0	At Admission: 3 Preoperative: 3 Day 1 postoperative: 3 Discharge (3 days postoperative): 4 Follow-up: 4
7	American Bulldog cross, 5 years 5 months, 50 kg	Preoperative: None Intra−/perioperative: Hypertension Postoperative: Regurgitation, cough, hematuria.	Score 1: 7 Score 2a: 0 Score 2b: 0	At Admission: 2 Preoperative: 2 Day 1 postoperative: 2 Discharge (8 days postoperative): 3 Follow-up: 4
8	Cross breed, 9 months, 12.4 kg	Preoperative: None Intra−/perioperative: None Postoperative: Regurgitation	Score 1: 6 Score 2a: 0 Score 2b: 0	At Admission: 4 Preoperative: 4 Day 1 postoperative: 4 Discharge (3 days postoperative): Normal Follow-up: Normal
9	Yorkshire Terrier, 1 year 8 months, 1.8 kg	Preoperative: None Intra−/perioperative: None Postoperative: None	Score 1: 5 Score 2a: 0 Score 2b: 0	At Admission: 2 Preoperative: 3 Day 1 postoperative: 3 Discharge (3 days postoperative): 3 Follow-up: 4
10	Cavapoo, 1 year 7 months, 7.6 kg	Preoperative: None Intra−/perioperative: None Postoperative: Mild dysphonia	Score 1: 6 Score 2a: 0 Score 2b: 0	At Admission: 4 Preoperative: 4 Day 1 postoperative: 4 Discharge (2 days postoperative): Normal Follow-up: Normal
11	Labradoodle, 1 year 5 months, 17.6 kg	Preoperative: None Intra−/perioperative: None Postoperative: Mild seroma on surgical wound	Score 1: 6 Score 2a: 0 Score 2b: 0	At Admission: 4 Preoperative: 4 Day 1 postoperative: 5 Discharge (5 days postoperative): Normal, mild left head tilt Follow-up: Normal
12	Dachshund, 6 months, 4 kg	Preoperative: None Intra−/perioperative: None Postoperative: Mild seroma on surgical wound, dysphonia	Score 1: 7 Score 2a: 1 Score 2b: 0	At Admission: 3 + vestibular signs Preoperative: 4 Day 1 postoperative: vestibular signs Discharge (4 days postoperative): vestibular signs Follow-up: Normal
13	Cavalier King Charles Spaniel, 8 months, 6.3 kg	Preoperative: None Intra−/perioperative: None Postoperative: cough, gaging, vomiting, diarrhea	Score 1: 4 Score 2a: 0 Score 2b: 0	At Admission: 4 Preoperative: 4 Day 1 postoperative: 4 Discharge (3 days postoperative): 4 Follow-up: 4
14	Chihuahua, 5 months, 1.5 kg	Preoperative: None Intra−/perioperative: hypoventilation, bradycardia under general anesthesia/tachycardia on recover Postoperative: None	Score 1: 4 Score 2a: 1 Score 2b: 0	At Admission: 2 Preoperative: 2 Day 1 postoperative: 3 Discharge (4 days postoperative): 3 Follow-up: 4
15	Chihuahua, 6 months, 3 kg	Preoperative: None Intra−/perioperative: None Postoperative: None	Score 1: 5 Score 2a: 0 Score 2b: 0	At Admission: 4 Preoperative: 4 Day 1 postoperative: 4 Discharge (4 days postoperative): 4 Follow-up: No data available
16	Chihuahua, 6 months, 1.6	Preoperative: None Intra−/perioperative: None Postoperative: Larynx edema and hypersalivation	Score 1: 5 Score 2a: 0 Score 2b: 0	At Admission: 4 Preoperative: 4 Day 1 postoperative: 4 Discharge (3 days postoperative): 4 Follow-up: deceased

**Table 2:** Long-term follow-up questionnaire (adapted from Microsoft Teams).

Long-term follow-up questionnaire	
Was your dog painful before the surgery?	9. Has your dog experienced recurrence of clinical signs?
Yes No	Yes No
Before the surgery, which clinical signs have worsened or additionally occurred?	10. If you answered YES to the previous question, please specify what clinical signs your dog has experienced
Loss of ability to walk unassisted Strength of gait/degree of limbs movements Scuffing of toenails None	
Is your dog currently able to walk unassisted? *	11. Is your dog currently on any medications? *
Yes, and with a normal gait Yes, but with a weak gait and scuffing of toenails No, but able to move all the limbs well with assistance No, little or no movement in any limbs	Yes No
Was your dog painful after the surgery on the first days after discharge?	12. If you dog is currently receiving medications, please specify the name, when and why these were started
Yes No	
Is your dog painful at the moment? *	13. How much activity is your dog currently performing? *
Yes No	Same as before the surgery Less than before the surgery Less than before the surgery but for reasons unrelated to the operation More than before the surgery
Has your dog experienced any complications since your last follow-up appointment with us?	14. Overall, how would you rate you dog’s current lifestyle? 1-very poor; 5-excellent *
Yes No	1 2 3 4 5
If you answered YES to the previous question, please specify what complications your dog has experienced	15. How would you say your dog’s current lifestyle is compared to before the surgery? *
	Improved The same Worsened
Since your last appointment with us, have your dog’s neurological signs …?	
Improved Remained static Deteriorated	

*For dogs that were deceased at the time the questionnaire was completed due to unrelated conditions, the questions phrased in the present tense were reformulated to refer to the period prior to death (e.g., ‘before the date of death’) these questions were modify as “before deceased date….”.

### 2.5 Long-term outcome

Data for this phase were collected via a standardized telephone questionnaire (Table 2). Long-term follow-up questionnaires were created using Microsoft Forms and distributed to eligible owners between May 2025 and November 2025. The median follow-up time for questionnaire responders was 4.3 years (range, 1 year – 9.7 years) postoperatively. The work described in this manuscript, single center retrospective study, involved the use of non-experimental (owned) animals. Established internationally recognized high standards (“best practice”) of veterinary clinical care of the individual patient were always followed after obtaining informed consent from the owners for each patient.

Participation was voluntary, and only owners of patients confirmed to be alive or those whose were deceased by unrelated conditions and the owner agreed to participate at the time of contact were invited to participate. For those deceased, the questions phrased in the present tense were reformulated to refer to the period prior to death (e.g., ‘before the date of death’). The questionnaire was designed to obtain subjective owner-reported outcomes pertaining to the patient’s functional recovery, specifically evaluating gait, muscular strength, cervical comfort, and overall quality-of-life. Cases for which long-term follow-up information could not be obtained were retained in the study and classified as “No data available”.

### 2.6 Statistical analysis

Descriptive statistics were employed for data analysis. All analyses were conducted using commercial spreadsheet software (Microsoft Excel, version 16.79.1; © Microsoft Corporation). Results are reported as medians, accompanied by the corresponding range of values.

## 3 Results

### 3.1 Signalment, initial presentation, and advanced imaging

A total of 16 dogs met the inclusion criteria (Table 1). Breeds included Chihuahua (*n* = 4), crossbreed (*n* = 2), English Pointer (*n* = 1), Pug (*n* = 1), Goldendoodle (*n* = 1), Toy Poodle (*n* = 1), Dalmatian (*n* = 1), Yorkshire Terrier (*n* = 1), Cavapoo (*n* = 1), Labradoodle (*n* = 1), Dachshund (*n* = 1), Cavalier King Charles Spaniel (*n* = 1). The median body weight was 11.9 kg (range 1.5 kg to 50 kg) and the median age at presentation was 2 years (range 5 months to 6 years of age). All dogs were reported to have neck pain at presentation. Neurological status at presentation using MFS grade showed grade 5 1/16, grade 4 7/16, grade 3 5/16 and grade 2 3/16. Although inclusion criteria allowed lesions affecting the C1 to C7 vertebrae, all included dogs presented with signs compatible with C1–C5 myelopathy apart from one. One dog had concurrent peripheral vestibular signs, including left head tilt, severe vestibular ataxia and left positional rotatory nystagmus with fast phase towards the right; secondary to traumatic tympanic bulla fracture and was therefore classified as having a multifocal neuroanatomical localization. The unstable cervical fracture was nevertheless considered the primary indication for surgical stabilization and inclusion of this case. Nine out of 16 dogs had cervical radiographs from which five out of nine were performed prior to the referral appointment. All dogs underwent CT examination, which confirmed cervical fractures in all cases. Five dogs (*n* = 5) additionally underwent an MRI scan. CT identified concurrent atlantoaxial subluxation in six dogs (*n* = 6), which was considered secondary to the C2 fracture. MRI identified Chiari-like malformation in one dog (*n* = 1), which was considered an incidental finding. In all cases, the cervical fracture was considered the primary cause of the neurological signs and the indication for surgical stabilization.

### 3.2 Surgical technique and post-surgery implant evaluation

On the day of surgery, 13/16 (81.3%) of dogs had maintained the same MFS score compared to their original presentation, 2/16 (12.5%) had improved and 1/16 (6.3%) had deteriorated (Table 1). Median time from the initial presentation in which CT scan was performed to surgery was 5.87 days (range 2 to 22 days). In most cases, 3D-PSGs were designed, manufactured and delivered within 2–7 days. The two longest intervals (15 and 22 days) were attributable to patient-specific clinical considerations. In one case, conservative management was initially pursued before surgery was decided upon due to lack of clinical improvement. In one other case, surgery was delayed to allow stabilization of concurrent traumatic injuries prior to cervical stabilization. Neck brace was placed in 9/16 dogs (56.3%). Of the nine dogs in which a neck brace was applied, seven remained neurologically stable until surgery, one improved and one showed a deterioration. Surgery was performed as described above. Screw grading based on the modified Zdichavsky classification was performed retrospectively by one of the authors using the immediate postoperative CT scan of each patient. A total of 90 pedicle screws were placed. Optimal pedicle screw placement (I) was obtained in 85/90 (94.4%) of screws while partial penetration of the medial pedicle wall (IIa) was present in 5/90 (5.6%). Full penetration of the medial pedicle wall (IIb) was not recorded in any case. Case-specific implant distribution and screw grading data are summarized in Supplementary Table S1.

### 3.3 Short-term outcome

Follow-up availability varied according to the outcome timepoint assessed (Figure 1). The day following the surgical procedure, 10/16 (62.5%) of dogs were categorized as having an unchanged neurological status (compared to their preoperative evaluation). Of the remaining dogs, 5/16 (31.3%) showed improvement, whereas 1/16 demonstrated a transient mild deterioration from MFS grade 5 to 4 which returned to grade 5 by the discharge. Complications were classified according to Cook et al. (21) Two dogs showed minor transient intraoperative or immediate perioperative (<12 h) complications (Table 1). Postoperative (>12 h) adverse events occurred in 10/16 (62.5%) dogs, varying in timing and severity; among these, eight dogs experienced minor complications. The most common adverse event was regurgitation (*n* = 4), followed by cough/retching/gagging (*n* = 3), surgical wound seroma (*n* = 2), dysphonia (*n* = 2), front paw edema (*n* = 1), hypersalivation (*n* = 1), constipation (*n* = 1), vomiting and diarrhea (*n* = 1), hematuria (*n* = 1) and laryngeal edema (*n* = 1).

One dog (*n* = 1) experienced a major complication characterized by persistent regurgitation requiring ongoing medical management.

One catastrophic complication occurred in a dog (*n* = 1) that was unexpectedly found deceased at home approximately 9 days after surgery (6 days after discharge). Forty-eight hours after discharge, the owner reported vomiting, anorexia and persistent cervical swelling, for which symptomatic treatment was instituted. At a telephone update 6 days after discharge, partial clinical improvement was reported, however, the dog was found deceased the following morning. Persistent laryngeal edema with suspected hypoventilation was considered the most likely cause of death.

The median duration of hospitalization was 4.2 days (range, 2–12 days). Hospitalization length for each individual case is provided in Table 1. Most dogs were discharged between 2 and 5 days following surgery. Two dogs (*n* = 2) required prolonged hospitalization (8 and 12 days, respectively) because of postoperative complications, including regurgitation and hematuria, which resolved with medical management before discharge.

At the time of discharge, the neurological grade remained unchanged in 6/16 dogs, whereas 10/16 dogs demonstrated an improvement in MFS. Of the dogs that improved, four were neurologically normal and free of cervical pain at discharge. One dog continued to exhibit a mild left head tilt, which had been present since admission, while another dog (*n* = 1) showed persistent vestibular signs attributed to concurrent traumatic middle/inner ear injury rather than the cervical spinal injury.

Short-term follow-up (4–6 weeks postoperatively) was available for 14/16 dogs. Follow-up information was unavailable for two dogs: one patient was lost to follow-up and one died 6 days after discharge, with suspected hypoventilation secondary to persistent laryngeal edema and coughing.

Among the 14 dogs with short-term follow-up, 11/14 underwent in-person neurological examination at the referral centre, while 3/14 were assessed remotely through telephone or email communication supplemented with owner-provided video recordings. Remote assessments were used when in-person evaluation was not feasible and were interpreted descriptively.

The majority of patients either showed neurological improvement (8/14) or remained at the same MFS (6/14) as recorded at the time of discharge, including one dog that was neurologically normal at discharge but exhibited mild intermittent cervical hyperesthesia during flexion at the follow-up visit following discontinuation of medication. In total, 2 out of 14 dogs reported cervical pain at follow-up, both cases being intermittent and elicited only during positional examination.

### 3.4 Long-term outcome

Long-term outcome information was obtained through a telephone questionnaire, and the results are summarized in Table 3. Twelve of 16 owners participated. Of these, nine dogs were reported to be alive at the time of data collection according to either their owner or primary veterinarian. The remaining three dogs had died from causes unrelated to the cervical fracture or due to age-related conditions.

**Table 3:** Results of the long-term follow-up questionnaire (adapted from Microsoft Teams).

Long-term follow-up questionnaire results			
1. Was your dog painful before the surgery?		9. Has your dog experienced recurrence of clinical signs?	
Category	No of dogs	Category	No of dogs
Yes	10 (83.3%)	Yes	5 (41.7%)
No	2 (16.7%)	No	7 (58.3%)
2. Before the surgery, which clinical signs have worsened or additionally occurred?		10. If you answered YES to the previous question, please specify what clinical signs your dog has experienced	
Category	No of dogs	Neck pain	
Loss of ability to walk unassisted	7 (58.3%)	Sporadic yelping Thoracic limb lameness and weakness Persistent mild head tilt	
Strength of gait/degree of limbs movements	3 (25%)		
Scuffing of toenails	1 (8.3%)		
None	1 (8.3%)		
3. Is your dog currently able to walk unassisted?*		11. Is your dog currently on any medications? *	
Category	No of dogs	Category	No of dogs
Yes, and with a normal gait	9 (75%)	Yes	3 (25%)
Yes, but with a weak gait and scuffing of toenails	3 (25%)	No	9 (75%)
No, but able to move all the limbs well with assistance	0		
No, little or no movement in any limbs	0		
4. Was your dog painful after the surgery on the first days after discharge?		12. If you dog is currently receiving medications, please specify the name, when and why these were started	
Category	No of dogs	Bedinvetmab, Acupuncture, hydrotherapy, gabapentin, meloxicam, paracetamol, Amantadine, amitriptyline, robenacoxib, methocarbamol.	
Yes	3 (25%)		
No	9 (75%)		
5. Is your dog painful at the moment?*		13. How much activity is your dog currently performing? *	
Category	No of dogs	Category	No of dogs
Yes	0	Same as before the surgery	8 (66.7%)
No	12 (100%)	Less than before the surgery	1 (8.3%)
		Less than before the surgery but for reasons unrelated to the operation	0
		More than before the surgery	3 (25%)
6. Has your dog experienced any complications since your last follow-up appointment with us?		14. Overall, how would you rate you dog’s current quality-of-life? 1-very poor; 5-excellent *	
Category	No of dogs	Category	No of dogs
Yes	4 (33.3%)	1	0
		2	0
		3	0
No	8 (66.7%)	4	1 (8.3%)
		5	11 (91.7%)
7. If you answered YES to the previous question, please specify what complications your dog has experienced		15. How would you say your dog’s current lifestyle is compared to before the surgery? *	
Surgical site infection, temporary voice loss, sporadic episodes of neck pain and sporadic episodes of muscle spasms on thoracic limbs		Category	No of dogs
		Improved	4 (33.3%)
		The same	8 (66.7%)
		Worsened	0
8. Since your last appointment with us, have your dog’s neurological signs …?			
Category	No of dogs		
Improved	12 (100%)		
Remained static	0		
Deteriorated	0		

Of the four dogs without long-term outcome data, two were lost to follow-up. One dog developed unrelated clinical deterioration during the data collection period and subsequently died; therefore, the owner did not participate in the telephone questionnaire. This patient had previously been reported as neurologically normal at the first follow-up examination, although chronic regurgitation persisted and was managed medically. Three and a half years later, worsening clinical signs, including regurgitation and coughing, prompted further investigation. CT scan confirmed implant stability, resolution of C2 fracture, and one broken screw without evidence of migration. Additional diagnostic evaluation identified tracheal collapse as a new and unrelated condition, which subsequently resulted in aspiration pneumonia and death. Another dog was unavailable for long-term assessment because the patient died before long-term follow-up could be performed.

At presentation 8/16 (50%) of dogs were not able to walk unassisted. All the dogs that participated in the questionnaire (*n* = 12) were reported to be ambulatory by their owners; however, abnormal gait (described as “weak gait and scuffing of toenails” in the questionnaire) was reported in 3/12 dogs.

All of the participating dogs were pain-free at the time of the questionnaire. Four of 12 dogs had postoperative complications including surgical site infection, temporary voice loss, sporadic episodes of neck pain and sporadic episodes of muscle spasms on thoracic limbs. All owners felt their dogs’ neurological signs had improved (12/12) since their last follow-up appointment at the referral centre where the spinal stabilization surgery had been performed. Five out of 12 had recurrence of the clinical signs that included neck pain, sporadic yelping, thoracic limb lameness or persistent head tilt.

Owners were asked to rate their dogs’ quality-of-life on a scale from 1 to 5 (1 = poor, 5 = excellent): 11/12 owners rated their dogs’ quality-of-life as 5/5 and 1/12 owners as 4/5. When asked if the animal’s current quality-of-life was considered improved, the same, or worsened in comparison with before the surgery, 4/12 owners replied “improved,” and 8/12 “the same,” none of them were reported as “worsened.”

## 4 Discussion

This retrospective descriptive study suggests that cervical spinal stabilization using 3D-printed patient-specific drill guides (3D-PSGs) is associated with high pedicle screw placement accuracy and favorable short- and long-term neurological outcomes in dogs with cervical vertebral fractures. Importantly, although the effect of surgical delay was not formally evaluated as an outcome in this study, the findings provide preliminary insight into the clinical course of dogs undergoing delayed surgical stabilization to allow for patient stabilization and 3D-PSGs planning and production. The low frequency of preoperative neurological deterioration observed in this cohort may suggest that, when combined with appropriate immobilization, monitoring and supportive care, delayed surgical stabilization does not necessarily result in worsening neurological status. These observations, including those related to preoperative immobilization and perioperative, postoperative and long-term complications, should be interpreted cautiously and warrant further investigation in larger cohorts.

Importantly, this study also addresses the clinical implications of delaying surgery to allow for patient stabilization, surgical planning including guide design and production, the role of preoperative immobilization, and the nature of perioperative, postoperative and long-term complications encountered in this population.

The accuracy of pedicle screw placement in this study was high, with 94.4% of screws graded as optimal and no instances of complete medial pedicle breach. This compares favorably with previously published veterinary studies evaluating 3D-PSGs and exceeds accuracy rates historically reported for freehand techniques. Given the small size of cervical pedicles and the proximity of critical neurovascular structures, the use of 3D-PSGs potentially contributed to the absence of severe implant-related complications and may support their use in cervical fracture stabilization. This is especially important in cases of cervical stabilization as a significant violation of the vertebral canal can be associated with severe neurological deficits and potentially fatal consequences. Furthermore, placement of pedicle screws in the cervical spine has been associated with high percentage of vertebral canal violation, 57 to 100% of vertebral canal (24) highlighting further the importance of surgical planning required in cervical stabilization.

The median time from presentation to surgery in this study was 5.87 days, reflecting the time required for advanced imaging, surgical planning, fabrication of the 3D-PSGs, and stabilization of patients with concurrent traumatic injuries. Prolonged hospitalization prior to surgery was therefore primarily related to the need for careful neurological monitoring, nursing care, and management of comorbidities rather than neurological instability alone.

The results may suggest that delaying surgery to allow for 3D-PSG production did not adversely affect neurological outcome. Most dogs remained neurologically stable during the preoperative period (13/16), and some reported neurological improvement prior to surgery (2/16). One dog experienced a prolonged delay of 22 days before surgery and remained neurologically stable, exhibiting only intermittent cervical pain (MFS grade 5). This observation supports the concept that, in selected cases without progressive neurological deficits, a controlled delay to allow for surgical planning may be clinically acceptable. The benefit in such surgical planning aside from increasing the safety of pedicle screw placement versus free hand, is an increased fixation strength by enabling bicortical screw trajectory and maximizing the screw diameter to pedicle width ratio to reduce the risks of construct failure.

Neurological deterioration prior to surgery was uncommon in this study and occurred in only one case. The deterioration was observed while a neck brace was in place, highlighting that external immobilization is not without potential complications and may not fully prevent instability-related neurological deficits. Conversely, neurological improvement prior to surgery was observed in two dogs, one of which had a neck brace applied. These improvements may reflect reduced cervical vertebral movement, resolution of spinal cord edema or effective analgesia.

Overall, the low rate of preoperative deterioration may indicate that delaying surgery, when combined with appropriate immobilization, supportive care and monitoring, does not necessarily increase neurological risk. However, this observation should be interpreted cautiously given the retrospective design and absence of a control group.

Neck braces were used in more than half of the dogs in this study (9/16). In one case, a dog that initially deteriorated subsequently improved neurologically following replacement of a poorly tolerated brace originally applied by the referring veterinarian. Although these observations may suggest a potential role for neck braces in maintaining preoperative stability in selected cases, no conclusions regarding their efficacy can be drawn from the present study.

Furthermore, neck brace use requires intensive nursing care and may be associated with complications including discomfort, stress, pressure sores or respiratory compromise. Careful application, regular reassessment, and adjustment or removal when poorly tolerated therefore remain important considerations. The potential role of neck braces as an adjunctive measure in the conservative management of cervical instability prior to definitive surgical stabilization may warrant further investigation in larger, controlled studies.

Consistent with previous reports, many dogs in this study sustained cervical fractures as a result of high-energy trauma and presented with concurrent injuries or comorbidities. These included additional orthopedic injuries or soft tissue trauma. The preoperative waiting period allowed time for stabilization of these conditions, optimization of anesthesia risk, and comprehensive surgical planning with the 3D-PSGs. This aspect may represent an underappreciated benefit of delayed surgery in complex trauma cases.

Concurrent imaging findings should be interpreted carefully. Atlantoaxial subluxation was identified in six dogs; however, this was considered a consequence of the traumatic C2 fracture rather than an independent concomitant condition, reflecting the instability created by disruption of the atlantoaxial articulation. In contrast, Chiari-like malformation was identified in one dog and was considered an incidental finding unrelated to the traumatic injury. Therefore, although incidental abnormalities may have contributed to the neurological presentation in isolated cases, the cervical fracture was considered the primary cause of the clinical signs and the indication for surgical stabilization in all dogs included in this study.

Postoperative complications ranged from minor, self-limiting adverse events to major and catastrophic complications. Minor complications were relatively common but were generally transient and resolved with medical management. The most frequently observed complications included regurgitation, coughing or gagging, dysphonia, and laryngeal edema, which are consistent with previously reported adverse events associated with ventral cervical approaches and prolonged anesthesia (25).

One dog experienced a major complication consisting of persistent regurgitation, resulting in a prolonged hospitalization (12 days). The regurgitation persisted after discharge but was successfully managed medically for approximately 3.5 years. The dog was later re-presented because of worsening regurgitation and coughing and was subsequently diagnosed with aspiration pneumonia, which ultimately resulted in death.

Another dog experienced a catastrophic complication and died approximately 7 days after discharge (9 days after surgery), with persistent laryngeal edema and suspected hypoventilation considered the most likely cause of death.

Overall, no intraoperative complications were observed. Postoperative adverse events ranged from minor, self-limiting complications (*n* = 9) to one major complication and one catastrophic complication. Although these severe cases resulted in death, the extent to which death was directly attributable to the surgical stabilization procedure remains uncertain, particularly in the dog that developed aspiration pneumonia several years after surgery. Nevertheless, these findings emphasize the importance of careful peri- and postoperative monitoring, particularly with respect to airway protection, swallowing function and gastrointestinal motility.

One dog demonstrated a persistent head tilt despite otherwise favorable neurological recovery. Although head tilt is most commonly associated with vestibular disease, recent study (26) has shown that cervical spinal or paraspinal pathology may also result in head tilt, potentially due to altered proprioceptive input, asymmetric cervical muscle tone, or nociceptive dysfunction. In that study, head tilt was reported in cases of cranial cervical myelopathy, typically involving the dorsal spinal cord segments from C1 to C3, as well as the spinal nerve roots or ganglia, and was hypothesized to result from disruption of vestibular pathways within the spinal cord. In the present case, the dog with persistent head tilt had a C2 fracture, which is consistent with these previously reported findings. The persistent head tilt was not associated with progressive neurological decline or reduced quality-of-life, suggesting a residual cervical origin rather than ongoing vestibular disease. Recognition of this phenomenon is clinically relevant and may help avoid misdiagnosis and unnecessary investigation.

Limitations of this study include its retrospective design, small sample size, incomplete follow-up data, reliance on subjective owner-reported long-term outcomes, and potential confounding effects of concomitant neurological conditions identified in one dog (Chiari-like malformation). Neurological grading was performed retrospectively and may have been influenced by incomplete documentation. Formal assessment of inter-observer and intra-observer agreement for screw position grading was not performed. Although all image evaluations were reviewed by a board-certified Neurology specialist, the reproducibility of the grading system was not formally quantified. In addition, the small sample size precluded meaningful subgroup or sensitivity analyses excluding dogs with concurrent neurological abnormalities.

In conclusion, in this study, dogs treated with cervical spinal stabilization using 3D-printed, patient-specific drill guides generally showed favorable outcomes in the dogs available for follow-up, with improvement in neurological status reported by all owners who participated in the long-term questionnaire (12/12). At the time of discharge, the neurological grade remained unchanged in 50% of dogs and improved in the remaining 50%, with half of the dogs demonstrating neurological normality and absence of cervical pain at discharge. In this retrospective cohort, the time required for design and manufacture of the drill guides was not associated with neurological deterioration in most dogs while awaiting surgery. Overall, in this cohort, the use of patient-specific 3D-printed drill guide was associated with accurate implant placement and generally favorable clinical outcomes during follow-up. Neurological recovery and favorable long-term functional outcomes were observed in the dogs available for follow-up. Postoperative complications, including severe adverse events in one dog, were observed and should be considered when interpreting these findings.

These results suggest that patient-specific 3D-printed drill guides represent a feasible surgical option for the stabilization of cervical vertebral fractures in dogs. However, larger prospective comparative studies are required to further evaluate the clinical performance and long-term outcomes of this surgical technique.

## Data Availability

The raw data supporting the conclusions of this article will be made available by the authors, without undue reservation.
